# Eating habits of a population undergoing a rapid dietary transition: portion sizes of traditional and non-traditional foods and beverages consumed by Inuit adults in Nunavut, Canada

**DOI:** 10.1186/1475-2891-12-70

**Published:** 2013-06-02

**Authors:** Tony Sheehy, Cindy Roache, Sangita Sharma

**Affiliations:** 1 School of Food and Nutritional Sciences, University College Cork, Cork, Republic of Ireland; 2 Department of Medicine, University of Alberta, #5-10 University Terrace, 8303 - 112 Street, Edmonton AB T6G 2T4, Canada

**Keywords:** Food portion sizes, Nutrition transition, Inuit, Nunavut, Canadian arctic

## Abstract

**Background:**

To determine the portion sizes of traditional and non-traditional foods being consumed by Inuit adults in three remote communities in Nunavut, Canada.

**Methods:**

A cross-sectional study was carried out between June and October, 2008. Trained field workers collected dietary data using a culturally appropriate, validated quantitative food frequency questionnaire (QFFQ) developed specifically for the study population.

**Results:**

Caribou, muktuk (whale blubber and skin) and Arctic char (salmon family), were the most commonly consumed traditional foods; mean portion sizes for traditional foods ranged from 10 g for fermented seal fat to 424 g for fried caribou. Fried bannock and white bread were consumed by >85% of participants; mean portion sizes for these foods were 189 g and 70 g, respectively. Sugar-sweetened beverages and energy-dense, nutrient-poor foods were also widely consumed. Mean portion sizes for regular pop and sweetened juices with added sugar were 663 g and 572 g, respectively. Mean portion sizes for potato chips, pilot biscuits, cakes, chocolate and cookies were 59 g, 59 g, 106 g, 59 g, and 46 g, respectively.

**Conclusions:**

The present study provides further evidence of the nutrition transition that is occurring among Inuit in the Canadian Arctic. It also highlights a number of foods and beverages that could be targeted in future nutritional intervention programs aimed at obesity and diet-related chronic disease prevention in these and other Inuit communities.

## Introduction

The traditional diet upon which Inuit of Arctic Canada, Alaska, Greenland and Chukotka (Russia) survived for millennia was based on a wide range of nutrient-dense foods obtained from the local environment, including wild game, marine mammals, fish, birds, and seasonal roots, stems, tubers, wild berries and edible seaweed [1-9]. Within the last fifty years, Inuit have come under increasing pressure to leave behind their traditional way of life and acculturate to the values of Western society [3,7,10]. This change in lifestyle has brought about a dramatic nutrition transition characterized by a decrease in the consumption of traditional foods and an increasing reliance on processed, store-bought foods imported from the south [7,11-20]. There is significant and valid concern for the health implications of consuming increased amounts of these fat- and sugar-rich foods [8,14].

Nunavut is the easternmost of three territories in Arctic Canada and consists of twenty-five remote and isolated communities spread across nearly two million square kilometers [21]. The population of Nunavut is approximately 33,000 [22] with some 85% of inhabitants being Inuit [21]. Despite having the youngest population in Canada [23], Nunavut is experiencing increasing rates of obesity and chronic non-communicable diseases. Obesity rates in Nunavut have increased from 23% in 1992 to 37% in 2004 [24]. Inuit have disproportionately higher rates of cancer compared with southern Canadians, including the highest incidence of salivary gland and lung cancers in the world and one of the highest rates of nasopharangeal cancer [25]. Age-standardized rates of cancer mortality (per 100,000 people) in 2007 were 340 in Nunavut compared with 166 for the general Canadian population [26]. Age-standardized mortality rates for diseases of the circulatory system (per 100,000 people) in 2000–2004 were 249 in Nunavut compared with 192 for the general Canadian population [27]. Life expectancy in Inuit-inhabited areas trails the Canadian average by more than twelve years [28]. Due to these high chronic disease prevalence rates and the remoteness of the communities in the territory, Nunavut’s health care system is under constant pressure due to the high cost of health service delivery [8]. Thus, from a health systems perspective, investing in chronic disease prevention is essential if the territory is to adequately and sustainably manage health care costs in the long term as its young population ages.

Obesity is a key target for nutritional interventions aimed at chronic disease prevention due to the fact that excess body weight is linked to a number of deleterious health effects including increased risk of coronary heart disease, ischemic stroke, hypertension, dyslipidemia, type 2 diabetes mellitus, joint disease, cancer, asthma, and a host of other chronic conditions [29]. Research suggests that over 86% of the variance in food intake among humans is due to factors in their immediate environment [30]. One important environmental factor that is believed to be contributing to the obesity epidemic is food portion size [31-40]. Portion sizes of virtually all foods and beverages prepared for immediate consumption have increased over the last few decades [31,32,41-43]. Increasing the amount of food [44-46] or caloric beverages [47] served on a given eating occasion results in an increase in energy intake, while serving larger food portion sizes for several days leads to a sustained increase in energy intake without any evidence of compensatory behaviour [48-50]. Nutritional interventions that focus on reducing portion sizes of energy-dense foods and increasing portion sizes of low-energy-dense foods such as soups, fruits and vegetables may represent one possible approach to moderating energy intake [36,51-53]. However, before such interventions can be attempted among Inuit, up-to-date information on the typical portion sizes of foods that are habitually consumed by this population is required. Previously, a culturally appropriate, validated quantitative food frequency questionnaire (QFFQ) was developed specifically for Inuit in Nunavut [54]. The objective of this study was to use this instrument to determine the portion sizes of traditional and non-traditional foods that are currently being consumed by Inuit adults in three remote communities undergoing a nutrition transition in Nunavut, Canada.

## Methods

The setting, recruitment methods and data collection procedures have been described in detail elsewhere [8]. In brief, a cross-sectional study was carried out in three communities in Nunavut, Canada, between June and October, 2008. Communities A, B and C were chosen to represent Inuit communities with varying population sizes, socioeconomic status and degrees of acculturation. Study participants were randomly selected using up-to-date community housing maps to ensure that those who had different proximities to stores and land for hunting were included. Residents aged <19 years and pregnant/lactating women were excluded due to their different nutritional requirements. The person in the household who was primarily responsible for shopping for and preparing foods was selected for interview to capture the kinds of foods commonly eaten within the population. Written informed consent was obtained from all participants.

Trained field workers collected dietary data using a culturally appropriate, validated QFFQ developed specifically for the study population [54,55]. Data on demographics, socioeconomic status, and heights and weights of participants for the calculation of BMI were also collected. The QFFQ contained 150 food items (65 meat, fish and poultry; 19 vegetables; 14 desserts and snacks; 13 fruits; 12 breads and cereals; 12 dairy; nine beverages; three alcoholic drinks; two sugar and sweetener products; one creamer product), of which 39 were traditional foods [8]. Participants were asked to report the frequency of consumption over a 30-day period by choosing from eight categories, which ranged from ‘never’ to ‘two or more times per day.’ For each food item, a separate question asked subjects to estimate how much they usually eat at one time. Three-dimensional food models (NASCO Company, 901 Jamesville Ave, Fort Atkinson, Wisconsin 53538) and household units (e.g., bowls, mugs, and spoons) were carefully chosen with input from local Inuit to best estimate the amount of foods and beverages consumed. Portion size was defined as the sizes in which foods are served at home and the units in which they are consumed in one sitting [56]. Data were examined by the project coordinator and if any set of data was incomplete the interviewer re-contacted the respondent to obtain the missing information. Upon completion of interviews, participants were given a CAD $25 gift certificate for a local store to recognize their contribution and thank them for their time.

For each of the foods and beverages listed on the QFFQ, the mean, standard deviation and median portion size in grams (for consumers only) were determined using SAS statistical software, version 9.2 (SAS Version 9.2, SAS Institute Inc., Cary, NC).

Institutional Review Board approval was obtained from the Committee on Human Studies at the University of Hawaii and the Office of Human Research Ethics at the University of North Carolina at Chapel Hill. The Nunavut Research Institute licensed the study.

## Results

In total, 211 Inuit adults (175 women and 36 men) participated in the study. Participants ranged in age from 19–89 years, with a mean (SD) age of 42.1 (15.0) years for men and 42.2 (13.2) years for women. The response rate was 69-93%, varying by community. There were 71 participants from Community A, 74 participants from Community B and 66 participants from Community C. Mean (SD) BMIs for participants from communities A, B and C were 29.4 (7.4) kg/m^2^, 29.4 (8.1) kg/m^2^ and 30.4 (7.7) kg/m^2^, respectively. At least two-thirds of participants in each community were either overweight or obese (BMI ≥ 25.0 kg/m^2^) according to WHO cut-off points [57].

The mean (SD) and median portion sizes (g) of traditional meats, traditional fish, and soups/stews consumed by Inuit adults in these three remote communities are shown in Table 1. Caribou was the most popular traditional meat, followed by muktuk (whale blubber and skin) and seal. Less than 20% of participants consumed other traditional meats such as goose, muskox, polar bear or ptarmigan and less than a third of participants consumed bone marrow or organ meats (e.g. heart, kidney, stomach, intestine). Mean portion sizes for individual items in this category ranged from 10 g for fermented seal fat (liquid) to 424 g for stir-fried caribou. Mean portion sizes for raw caribou and raw seal were 274 g and 195 g, respectively. With regard to fish, more than half of the participants consumed Arctic char; other traditional fish such as trout or white fish were consumed by fewer participants. Mean portion sizes for fish items ranged from 83 g for battered and/or fried fish to 370 g for raw Arctic char. Soups or stews were consumed by the majority of participants. Caribou soup or stew was consumed by three-quarters of participants with an average portion size of 475 g. Fish soup or chowder was consumed by one-third of participants, with an average portion size of 466 g.

**Table 1:** Mean (SD) and median portion sizes (g) of traditional meats, poultry, fish and soups/stews consumed by Inuit adults in three remote communities in Nunavut, Canada

***Food item***	***Consumers *(%)**	***Portion size *( *g *)**		
		**Mean**	**SD**	**Median**
Traditional meats/poultry				
Bone marrow, any	31.3	34	22	32
Caribou, aged	2.4	251	138	279
Caribou fat, hard	38.4	62	52	32
Caribou, boiled, baked or roast	82	191	107	149
Caribou, dried	60.2	201	166	153
Caribou, fried, not incl. stir fry	55.9	96	59	58
Caribou, raw	66.4	274	205	186
Caribou, stir fried	34.1	424	190	428
Goose, baked, boiled or roasted	19.4	88	75	82
Heart or kidney, any	24.6	389	267	200
Liver, any, not incl. seal	12.3	124	74	110
Muktuk	48.3	217	178	194
Muskox, boiled	10	161	106	173
Muskox, fried	5.7	143	73	143
Muskox, fat	3.3	39	30	32
Polar bear, boiled	6.2	169	136	149
Ptarmigan	4.3	277	261	119
Seal, cooked	37.9	184	96	159
Seal fat, fermented or fresh, hard	12.3	36	28	26
Seal fat, fermented, liquid	4.3	10	4	10
Seal liver	24.6	145	132	88
Seal, raw, not incl. liver	12.3	195	143	167
Stomach or intestine, any	12.3	133	109	101
Traditional fish				
Char, smoked	14.7	95	66	62
Char, boiled	53.1	256	112	209
Char, dried	60.7	86	49	93
Char, raw	63	370	198	261
Fish head, large	9	269	102	227
Fish head, medium	21.8	151	73	142
Fish head, small	7.6	92	26	89
Fish, baked	28	204	143	185
Fish, battered and/or fried	42.7	83	43	84
Trout, baked or broiled	15.2	219	94	201
Trout, dried	21.8	179	127	165
Trout, raw	13.7	227	110	237
White fish, dried	7.1	105	71	110
White fish, raw	7.1	261	105	237
Soups/stews				
Beef stew, homemade or canned	41.7	360	146	368
Caribou soup or stew	75.8	475	199	405
Char or clam chowder or any fish soup	34.6	466	171	452
Mushroom or vegetable soup	49.3	349	121	381
Soup, any, with beef, ham, chicken, duck, goose	53.6	419	160	418

The mean (SD) and median portion sizes (g) of fruits and vegetables consumed by this Inuit population are shown in Table 2. About three-quarters of participants consumed apples, bananas, grapes or oranges and approximately 60% consumed canned fruit or fruit cocktail. However, most other fruits on the QFFQ were consumed by less than one-third of participants. The mean portion sizes for fruits ranged from 36 g for grapes to 260 g for fresh fruit salad. With regard to vegetables, slightly more than half the participants consumed carrots, corn, or frozen vegetables, but consumption of other vegetables was limited, both in terms of variety and amount. Mean portion sizes for vegetables ranged from 78 g for corn to 177 g for salad.

**Table 2:** Mean (SD) and median portion sizes (g) of fruits and vegetables consumed by Inuit adults in three remote communities in Nunavut, Canada

***Food item***	***Consumers *(%)**	***Portion size *( *g *)**		
		**Mean**	**SD**	**Median**
Fruit				
Apple	73.9	145	56	138
Banana	84.4	156	68	136
Blueberries, raspberries, blackberries, any other	41.7	99	43	104
Dried fruits incl. raisins	42.2	108	74	87
Fruit or fruit cocktail, any, canned in syrup	59.7	194	78	188
Fruit salad, fresh	14.7	260	136	183
Fruit, frozen, incl. peaches, strawberries, blueberries	29.9	186	71	164
Grapes	74.4	36	25	24
Kiwi	25.6	125	72	91
Mango	6.6	152	59	155
Orange	74.4	146	64	131
Peaches and nectarines	13.7	133	52	117
Strawberries	29.9	115	52	108
Vegetables				
Carrot, eaten alone	54.5	159	229	114
Corn	55.5	78	28	73
Corn on the cob	28.4	159	72	146
Fresh vegetables, other	29.4	97	41	118
Frozen vegetables, any, incl. mixed	58.3	92	36	117
Salad in a bowl	37.9	177	59	207
Tomatoes, canned	14.2	167	76	183
Vegetables, canned, any	17.5	97	50	73

The mean (SD) and median portion sizes (g) of cereal-based foods (including breads, pancakes, breakfast cereals, porridges, noodles, macaroni and rice) and potatoes consumed by Inuit adults in this study are shown in Table 3. More than 85% of participants consumed fried bannock, whereas baked bannock was consumed by only 19.4% of participants. Mean portion size of fried bannock was higher than that of baked bannock (189 g vs 166 g, respectively). White bread was consumed by 87.7% of participants, whereas only 57.8% of participants consumed whole wheat bread. Mean portion sizes for white and whole wheat bread were more or less the same (approximately 70–74 g). Breakfast cereals were consumed by slightly less than half of the participants. Mean portion sizes for sweet cereals were higher than those for low-sugar cereals (46 g vs 29 g, respectively). Noodles, macaroni, potatoes, potato products and especially rice were consumed by a large percentage of participants. Mean portion sizes for these food items ranged from 97 g for hash browns/potato patties/French fries to 475 g for noodles.

**Table 3:** Mean (SD) and median portion sizes (g) of breads/pancakes, cereals/porridges, noodles/macaroni/rice and potatoes consumed by Inuit adults in three remote communities in Nunavut, Canada

***Food item***	***Consumers *(%)**	***Portion size *( *g *)**		
		**Mean**	**SD**	**Median**
Breads/pancakes				
Bannock, baked	19.4	166	127	139
Bannock, fried	85.8	189	116	149
Pancakes or waffles, incl. Eggo waffles	51.7	88	32	91
White bread, incl. toast, sandwiches, rolls and bagels	87.7	70	30	60
Whole wheat bread	57.8	74	25	67
Cereals/porridges				
Low sugar cereals (e.g. Corn Flakes, Rice Krispies, Cheerios)	49.3	29	10	28
Porridge, home-made	25.6	186	91	142
Quaker Oats or porridge in a package	42.7	254	103	189
Sweet cereals, incl. Frosted Flakes or Honey Nut Cheerios	43.1	46	17	51
Noodles/macaroni/rice				
Macaroni and cheese or Kraft Dinner	67.3	300	111	343
Noodles	61.1	475	212	450
Rice, any	85.3	148	76	131
Potatoes				
Hash browns or potato patties or French fries	67.8	97	61	77
Potato salad	19.4	158	84	175
Potato, baked or boiled	60.7	141	60	119
Potato, mashed, incl. instant	66.8	125	54	103

The mean (SD) and median portion sizes (g) of dairy products, eggs, and non-traditional meats and meat products consumed by Inuit adults in this study is shown in Table 4. Milk (2% fat) was consumed by more than half the participants, although other milks (i.e. skim, 1% fat, or whole milk) were consumed by less than one-tenth of participants. About one-fifth of participants consumed milkshake, hot chocolate or canned/evaporated milk products. Portion sizes for milks were broadly similar at approximately 343–380 g. Hard cheese and eggs (chicken or duck) were consumed by three-quarters of participants. The most popular non-traditional meat products were hot dogs/wieners/sausages, which were consumed by 75.8% of participants with a mean portion size of 115 g. At least half of participants reported consuming beef hamburgers, beef steak, pork chops, pork or beef ribs, chicken legs and chicken wings, while other processed meat products including salami/bologna, pepperoni, jerky and canned meats were also widely consumed.

**Table 4:** **Mean** (**SD**) **and median portion sizes** (**g**) **of dairy products**, **eggs**, **and non**-**traditional meats and meat products consumed by Inuit adults in three remote communities in Nunavut**, **Canada**

***Food item***	***Consumers *(%)**	***Portion size *( *g *)**		
		**Mean**	**SD**	**Median**
Milk				
Milk, 1 % or skim	8.1	371	182	259
Milk, 2 %	57.3	343	224	259
Milk, Carnation, cream, half and half or carnation cream	21.3	68	73	40
Milk, whole	3.8	354	316	259
Milkshake or hot chocolate	22.7	380	193	283
Cheese				
Cream cheese, any	17.1	24	32	15
Hard cheese incl. Kraft cheese slices	77.7	54	36	30
Eggs				
Chicken or duck eggs	75.8	93	26	85
Goose eggs	14.7	218	148	144
Swan eggs	2.4	202	79	144
Beef/pork				
Beef hamburgers	56.9	154	65	101
Beef steak, not incl. stir fry	51.7	158	57	157
Beef, stir fried	19.9	349	129	425
Meat pie	12.8	206	127	170
Pork chops	59.2	116	49	86
Pork or beef ribs	53.1	120	58	108
Pork roast	12.8	84	50	58
Sloppy Joe	7.1	195	71	146
Spaghetti with ground beef or ground muskox, or beef ravioli	70.1	354	122	373
Processed meats				
Bacon, fried	60.2	29	15	24
Beef or muskox jerky	46.9	39	21	39
Bologna, salami	52.1	52	26	56
Ham	30.8	92	60	72
Hot dogs, wieners or sausages	75.8	115	51	107
Klik or other canned meat	65.4	70	57	45
Pepperoni sticks	43.6	72	40	56
Chicken/turkey				
Chicken breast, baked, boiled or roasted	31.3	291	158	225
Chicken breast, fried, incl. KFC	14.7	140	51	124
Chicken leg, baked, boiled or roasted	52.1	219	116	226
Chicken leg, fried, incl. KFC	22.7	216	111	223
Chicken nuggets or popcorn chicken	41.2	103	39	96
Chicken wings	55.5	41	18	36

The mean (SD) and median portion sizes (g) of energy-dense, nutrient-poor foods (e.g. chips, popcorn, desserts, candies/candy bars, cookies and crackers) and beverages consumed by this population of Inuit adults are shown in Table 5. More than three-quarters of participants reported consuming potato chips and pilot biscuits, while at least half the participants consumed cakes/muffins, chocolate, cookies and crackers. Mean portion sizes for foods in this category ranged from 13 g for popcorn to 170 g for cheesecake. More than three-quarters of participants reported consuming tea, coffee and regular non-alcoholic beverages (regular pop and sweetened juices with added sugar), whereas less than 20% consumed diet or sugar-free beverages. Regular pop was consumed in larger portion sizes than diet pop (663 g vs 459 g, respectively), and sweetened juices with added sugar were consumed in larger portions than sugar-free juices (572 g vs 514 g, respectively). Alcoholic beverages were consumed by less than one-third of participants.

**Table 5:** Mean (SD) and median portion sizes (g) of chips/popcorn, desserts/candies/cookies, crackers and non-alcoholic and alcoholic beverages consumed by Inuit adults in three remote communities in Nunavut, Canada

***Food item***	***Consumers *(%)**	***Portion size *( *g *)**		
		**Mean**	**SD**	**Median**
Chips/popcorn				
Popcorn	42.7	13	6	12
Potato chips	83.4	59	26	60
Desserts/candies/candy bars/cookies				
Cake or muffin, any	59.7	106	52	117
Cheesecake or similar	6.6	170	65	134
Chocolate bar, any kind	60.7	59	28	52
Cookies incl. Oreos, Oatmeal	50.2	38	21	30
Hard candy, any	42.2	21	14	18
Pie, blueberry, apple, cherry	29.4	154	78	118
Crackers				
Crackers incl. Cream Crackers, Premium Plus	56.9	46	38	31
Crackers incl. Ritz, Wheat Thins or sesame snacks	29.9	20	15	15
Pilot biscuits, any kind	75.8	59	40	51
Regular beverages				
Juice sweetened, any kind, with added sugar	77.7	572	414	527
Pop, regular	82	663	706	372
Tea/coffee				
Coffee	81.5	1075	984	752
Tea, any hot tea	83.9	786	697	502
Diet/sugar-free beverages				
Pop, diet	17.1	459	226	355
Sugar-free juices	15.2	514	403	507
Alcohol				
Beer or coolers, any kind	15.6	1826	1189	1426
Liquor incl. rum, whiskey, vodka or gin	30.8	330	418	158
Wine, any	8.1	492	444	374

## Discussion

The present study provides up-to-date information on portion sizes of traditional and non-traditional foods and beverages as consumed by Inuit adults in three remote communities in Nunavut in the Canadian Arctic. Caribou, muktuk and Arctic char were the most widely consumed traditional foods. However, sugar-sweetened beverages and energy-dense, nutrient-poor foods (e.g. potato chips, pilot biscuits, cakes, chocolate, cookies and crackers) were widely consumed also. A number of factors may have contributed to the decline in traditional food consumption that has been reported among Arctic indigenous peoples in recent years; these include lack of time for hunting due to increased involvement in the wage economy, high cost of hunting equipment, ammunition and fuel, a decline in communal food sharing networks, concerns about food supply contamination by organochlorines and heavy metals, and reduced animal populations and changing migration patterns due to climate change [7,8,58].

The present study also revealed that apart from apple, banana, oranges and grapes, which were consumed by roughly three-quarters of participants, fruit and vegetable consumption by Inuit participants in these 3 communities was generally low. The landscape in this region is tundra and is covered in snow for most of the year; hence there is an almost total reliance on fruits and vegetables that are grown elsewhere and transported to the communities primarily by air freight [7]. Mean daily temperatures are below 0°C for approximately nine months of the year and below −30°C for about four months of the year [59]. Transportation and preservation of fresh fruits and vegetables under these conditions is difficult and costly; thus, the produce that is available in the stores is often of poor quality and prohibitively expensive [7,8]. Basic nutrition education and healthy dietary skills may also be a barrier to fruit and vegetable consumption in these communities [7,19].

Sweetened beverages were consumed by over three-quarters of participants in the present study and the average portion sizes were large. Juices consumed by this population are mainly high sugar beverages such as Kool-Aid™ (Kraft Foods Inc., Northfield, IL, USA) or Tang™ (Kraft Foods Inc., Northfield, IL, USA) [17]. The average portion size for sweetened juices with added sugar was 572 g, which in terms of caloric intake would provide some 879–1130 kJ (210–270 kcal) [60]. Similarly, the average portion size for regular pop was 663 g (equivalent to about two standard 330 ml cans), which would provide about 1151–1339 kJ (275–320 kcal) [60]. By comparison, in the United States, the average portion size of sweetened beverages consumed per eating occasion increased from 386 g to 595 g between 1977 and 1996 [61]. Energy from caloric beverages is poorly regulated and therefore can add excess calories to daily energy intake [47,62]. Sugar-sweetened beverage intake is a significant contributor to weight gain and can lead to increased risk of type 2 diabetes mellitus and cardiovascular disease [63]. Recently, the American Heart Association issued a scientific statement recommending that in order to achieve and maintain healthy weights and decrease cardiovascular risk while at the same time meeting essential nutrient needs, most American women should eat or drink no more than 100 calories per day from added sugars, and most American men should eat or drink no more than 150 calories per day from added sugars [64]. This suggests that Inuit consumers in these communities would need to reduce portion sizes of sugar-sweetened beverages by a very considerable margin and to replace them with water, tea or coffee (provided that caloric sweeteners and whiteners are used sparingly) or with diet- or sugar-free varieties of the same products.

In the present study, potato chips were consumed by over four-fifths of participants, with an average portion size of 59 g; this would provide roughly 1151–1381 kJ (275–330 kcal), depending on brand [60]. Likewise, chocolate was consumed by almost 60% of participants, and the energy provided by an average portion (59 g) would be around 1046–1339 kJ (250–320 kcal), depending on brand [60]. Cakes and muffins were consumed by some 60% of participants and the average portion size was 106 g. In terms of energy content, commercial muffins provide approximately 1159–1360 kJ (277–325 kcal)/100 g, while commercial cake varieties (e.g. fruit cake, sponge cake, chocolate cake, coffee cake, white cake) provide some 1205–1736 kJ (288–415 kcal)/100 g [60]. Certain other types of refined grain products (e.g. spaghetti, macaroni and noodles) also contributed substantial amounts of energy to consumers in this population due to the large portion sizes consumed. On the other hand, the popularity of soups among participants could be exploited because soups have a high satiety value and elicit strong dietary compensation [65]; eating a low energy-dense soup as a pre-load before a test meal resulted in a 20% reduction in total energy intake at the meal with no significant effect on hunger or satiety ratings [52]. Thus, the present study has highlighted a number of different types of foods and beverages that could be targeted in future nutritional intervention programs aimed at obesity and diet-related chronic disease prevention in these and other Inuit communities.

Due to its geographic remoteness, the cost of treating chronic disease in Nunavut is extremely high as a result of the necessity to transport patients to southern cities for medical assessment and treatment [8]. This situation will get markedly worse if, as is predicted, the global obesity epidemic that is currently happening is followed by an epidemic of type 2 diabetes and other cardiovascular disease risk factors [66,67]. Recently, the American Heart Association endorsed the concept of ‘primordial prevention’ for cardiovascular disease prevention [68]. This approach is based on evidence from the Framingham Heart Study cohort showing that compared with individuals with ≥2 major risk factors, those individuals who had maintained a profile of ideal cardiovascular risk factor levels from young adulthood into middle age had greatly reduced lifetime CVD and non-CVD mortality rates, thereby resulting in an additional 10 years’ longevity [69]. Capewell et al. [70] demonstrated that if the majority of the US population reached middle age with this ideal phenotype, more than 90% of expected coronary heart disease deaths might be prevented. However, it was also argued that to bring about such a change would require an environment that supports health, rather than, as now, promoting obesity, hypertension, diabetes and inactivity [71]. For Nunavut, the implication of these findings is that investment in programs and public health policies that prevent the development of adverse CVD risk factors in its young population could be expected to yield substantial returns in the long term in the form of greatly reduced medical costs and increased longevity and quality of life for its citizens. On the other hand, failure to do so would place its health care system under an increasingly unsustainable burden as its young population ages.

The results of the present study were obtained as part of Healthy Foods North (HFN), a community-based, culturally-appropriate, multi-institutional chronic disease prevention program that has worked at the individual, household, community and institutional level to improve diet and increase physical activity among Inuit in Nunavut [8,55]. These data will be useful in developing other nutritional intervention programs designed to reduce dietary risk factors for obesity and chronic diseases in Inuit populations.

A major strength of the present study is the fact that the QFFQ was developed specifically for this Inuit population and thus contains the complete list of foods commonly consumed by them. Also, portion sizes were assessed using three dimensional food models and household units that were chosen with the input of local Inuit residents. The study does, however, have some limitations. Firstly, men were not as well represented as women since the intention was to target the family member who was primarily responsible for purchasing and preparing foods. Therefore, our ability to generalize these results to Inuit men is limited. Secondly, there may have been recall biases among the participants when they reported foods and beverages consumed in the last 30 days. Thirdly, the study only captured summer and autumn consumption and, consequently, did not account for seasonal variability, especially during the winter months. Finally, it should not be assumed that data collected in these three specific communities can be generalized to all Inuit populations.

## Conclusions

This study presented contemporary data on portion sizes of traditional and non-traditional foods and beverages consumed by Inuit in three remote communities in Nunavut, Canada. Caribou, muktuk and Arctic char were the most widely consumed traditional foods. However, energy-dense, nutrient-poor foods and sugar-sweetened beverages were widely consumed as well. Based on observed portion sizes and rates of consumption among participants, a number of foods and beverages were highlighted that could be targeted in future nutritional intervention programs aimed at obesity and diet-related chronic disease prevention in these and other Inuit communities. The results of this study may also be useful for nutrition education and for monitoring the ongoing nutrition transition among Inuit in the Canadian Arctic.

## Competing interests

The authors declare that they have no competing interests.

## Authors’ contributions

TS participated in manuscript drafting and editing, CR oversaw all field activities and data collection, SS conceptualized and participated in manuscript drafting, design, and critical review. All authors read and approved the final manuscript.
